# Calibration of solar pyranometers for accurate solar irradiance measurements: A data set from the pre- and post-calibration process

**DOI:** 10.1016/j.dib.2025.111402

**Published:** 2025-02-19

**Authors:** H. Alkhatib, B. Norton, D.T.J. O'Sullivan, P. Lemarchand

**Affiliations:** a School of Mechanical Engineering, Technological University Dublin, Dublin, Ireland; b Dublin Energy Lab, Technological University Dublin, Dublin, Ireland; c MaREI, the SFI Centre for Energy, Climate and Marine, Dublin, Ireland; d Sustainability Intelligence, Technological University Dublin, Dublin, Ireland; e IERG, School of Engineering, University College Cork, Cork, Ireland; f IERC, International Energy Research Centre, Tyndall National Institute, University College Cork, Cork, Ireland

**Keywords:** U-value, Energy audit, Thermal performance, Building efficiency, Insulation

## Abstract

This study outlines the calibration of five solar pyranometers following ISO 9847 standards to improve the accuracy of solar irradiance measurements. The calibration process involved data collection, consistency checks, and calculations to determine calibration factors. Post-calibration results demonstrated reduced systematic errors and more accurate irradiance readings. The resulting data set reports on the entire calibration process, including pre- and post-calibration irradiance measurements, which enhances the reliability of solar energy measurements for future applications. The calibrated pyranometers were then used to measure vertical, horizontal, and ground-reflected solar irradiance, including diffuse radiation.

Specifications Table

**Table:** 

Subject	Energy
Specific subject area	Renewable Energy, Sustainability and the Environment
Type of data	Raw, Table and Figures
Data collection	The data was collected during the calibration of five solar pyranometers, following ISO 9847 standards. Solar irradiance measurements were taken at 5-minute intervals over several days, both before and after the calibration, along with temperature data to monitor environmental conditions. Solar irradiance was recorded using all pyranometers, with comparisons made against a reference pyranometer. The data demonstrates a reduction in measurement errors post-calibration. Calibration factors for each pyranometer were calculated to improve the accuracy of future solar irradiance measurements.
Data source location	The data was collected at an outdoor solar testing facility. The pyranometers were mounted in a horizontal position to capture solar irradiance data. Temperature readings were recorded using a weather station. The calibration process followed ISO 9847 standards. Data was logged using a DL2e data logger for analysis before and after calibration.
Data accessibility	Alkhatib, Hani; lemarchand, philippe; Norton, Brian; O' Sullivan, Dominic (2024), “Calibration of Solar Pyranometers for Accurate Solar Irradiance Measurements: A Data Set from the Pre- and Post-Calibration Process.”, Mendeley Data, V1, doi: 10.17632/8ptkfx254m.1 Repository name: **Calibration of Solar Pyranometers for Accurate Solar Irradiance Measurements: A Data Set from the Pre- and Post-Calibration Process.** Data identification number: doi: 10.17632/8ptkfx254m.1 Direct URL to data: https://data.mendeley.com/datasets/8ptkfx254m/1 Instructions for accessing these data: **Non**
Related research article	**Non applicable.**

## 1 Value of the Data

• The dataset provides a comprehensive overview of the calibration process for solar pyranometers, enhancing the accuracy of solar irradiance measurements.
• It supports the improvement of solar energy monitoring systems by offering detailed calibration factors for multiple pyranometers.
• The dataset highlights the impact of systematic calibration, showing significant reductions in measurement errors post-calibration.
• By comparing pre- and post-calibration readings, the data offers insights into the performance and reliability of pyranometers under various environmental conditions.
• This dataset is essential for researchers and engineers working on optimizing solar energy systems and improving irradiance measurement techniques.

## 2 Background

Both the wall and window elements of a facade can be engineered to (i) harness solar energy for photovoltaic electricity generation, heating, inducing ventilation and daylighting, (ii) provide varying levels of thermal insulation and (iii) store energy [[1], [2], [3]]. As a façade may need to provide each of these attributes to differing extents at particular times, achieving their optimal performance requires knowledge of the energy losses throughout the building. These, together with thermally inefficient materials and systems, lead to 40% of global energy consumption [4,5] being used to heat, cool, light and ventilate buildings [[6], [7], [8]].

Previous studies on solar irradiance measurements have often lacked precision due to uncalibrated instruments, leading to inaccuracies in solar energy data. This dataset addresses this gap by providing a comprehensive analysis of pyranometer calibration, following ISO 9847 standards, to ensure reliable measurements. By comparing pre- and post-calibration data from multiple pyranometers, this study highlights a significant reduction in measurement errors, enhancing the accuracy of solar energy assessments. These findings are invaluable for researchers and engineers working to improve solar systems, environmental monitoring, and renewable energy applications. This study sets the foundation for improved calibration practices that can drive efficiency in future solar energy projects and advance renewable energy technologies [[9], [10], [11], [12], [13]].

## 3 Data Description

The dataset includes solar irradiance measurements collected using five calibrated pyranometers, with data recorded at 5-minute intervals over several days. It captures solar irradiance before and after calibration, along with temperature readings to ensure environmental monitoring. The calibration process followed ISO 9847 standards and involved calculating calibration factors for each pyranometer. To improve data accuracy, systematic checks were performed, and inconsistencies were identified and addressed. Pre- and post-calibration comparisons showed significant improvements in measurement precision, offering more reliable solar irradiance data for future solar energy applications. The dataset includes the pyranometer output in voltage (V) along with a calibration factor to convert the voltage into radiation measured in watts per square meter (W/m²). Different pyranometer models can use the same ISO standard for calibration.

## 4 Experimental Design, Materials and Methods

**Fig. 1:**
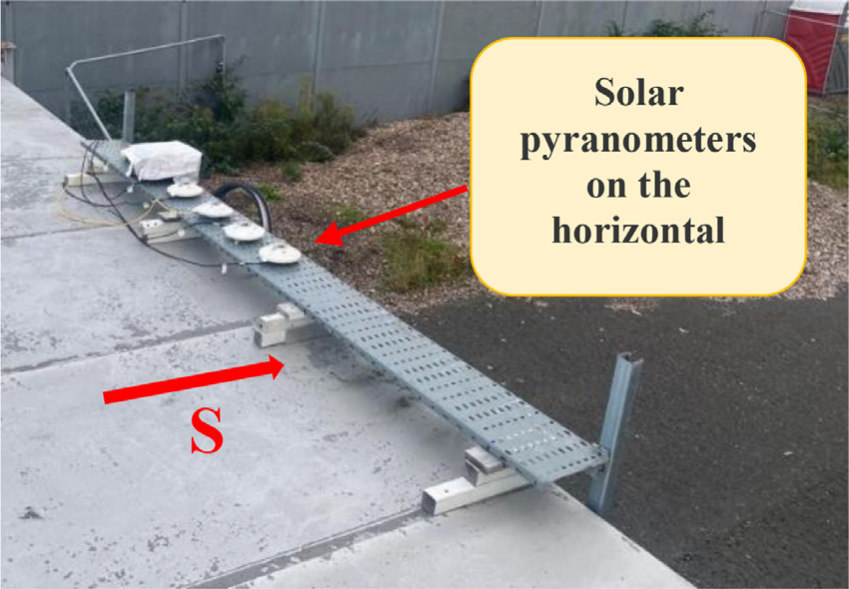
Solar pyranometers positioning during calibration.

**Fig. 2:**
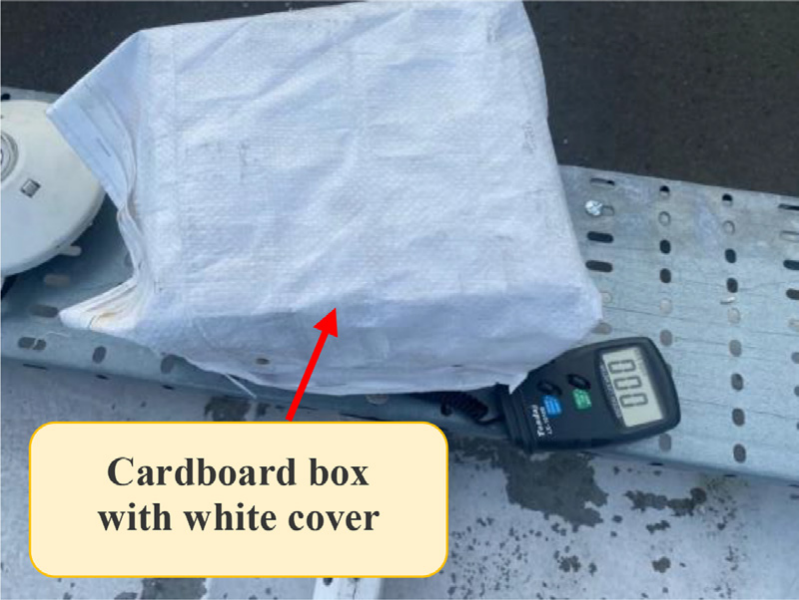
Box used in solar pyranometer calibration.

**Fig. 3:**
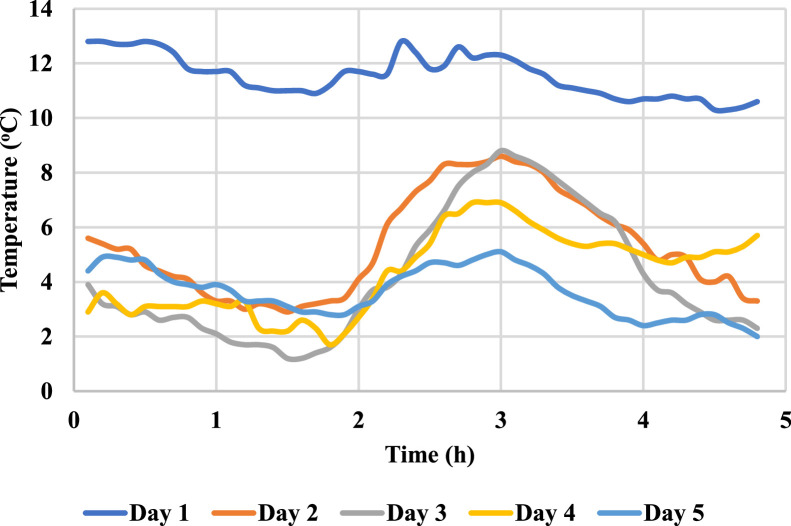
Outdoor air temperature during solar pyranometer calibration.

**Fig. 4:**
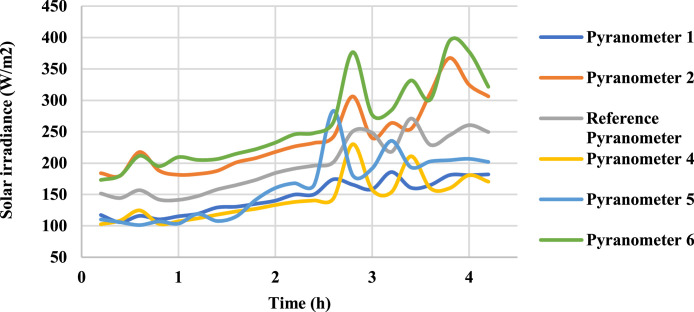
Solar irradiance before calibration

**Fig. 5:**
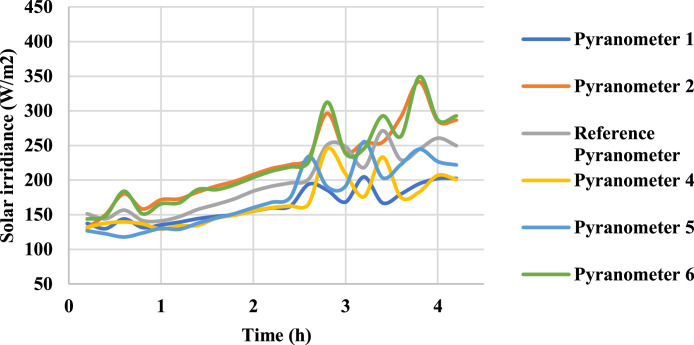
Solar irradiance after calibration.

**Fig. 6:**
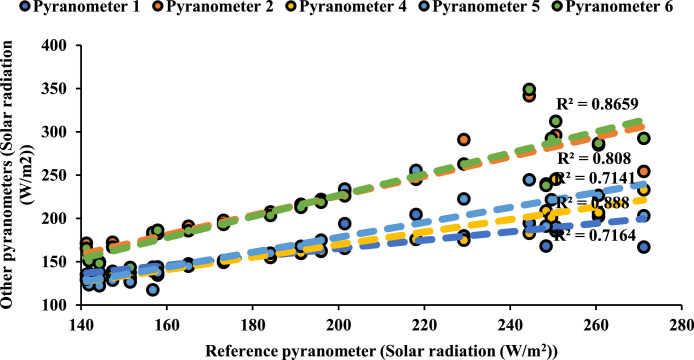
Relationship between reference pyranometer and other pyranometers before calibration.

**Fig. 7:**
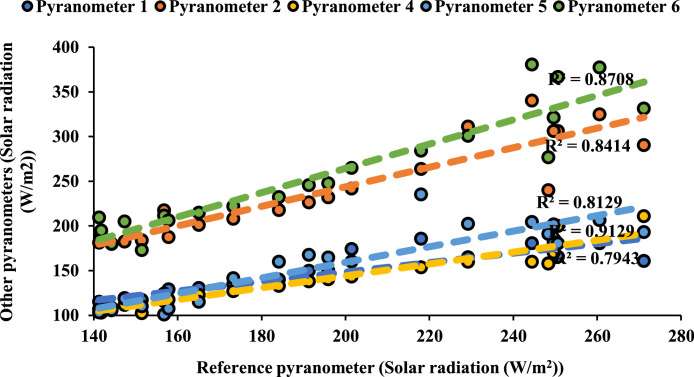
Relationship between reference pyranometer and other pyranometers after calibration.

Following ISO 9847 [14], and using a reference pyranometer calibrated by the manufacture company (Pyranometer 3 in Table 1), five main steps were taken to calibrate the five solar pyranometers: (i) all five solar pyranometers were mounted horizontally as shown in Fig. 1, (ii) readings were taken over 5 sunny days at 5-minute intervals using a “DL2e” data logger [15], Measurements were specifically taken on sunny days as specified by ISO 9847 standards to ensure consistent solar irradiance and minimize the influence of cloud cover, which can significantly alter the accuracy of solar radiation data. (iii) A small box (shown in Fig. 2) was placed over each solar pyranometer four times a day to determine if the minimum reading remains consistent over the 5 days, (iv) outside air temperatures (shown in Fig. 3) were recorded using the weather station and (v) calculations were done according to the ISO 9847 standard to calculate for each solar pyranometer (i) solar curves and (ii) calibration factors (shown in Table 1). Figs. 4 and 5 show the solar irradiance readings on 21/Nov/2021 pre and post calibration, respectively. It can be observed that the differences between the readings were less after the calibration process. Figs. 6 and 7 show the relation between the reference pyranometer and other pyranometers before and after the calibration, respectively. It can be observed that systematic errors were reduced after calibration. To optimize the calibration methodologies of ISO 9847 for non-standard conditions such as high altitudes or extreme weather environments, several modifications can be made to enhance pyranometer performance such as adjusting the calibration coefficients and temperature compensation or the enhanced sensor shielding. ISO 9847 standards reduce calibration errors in pyranometers by standardizing setup and procedures, implementing temperature compensation, and enforcing quality control checks. These measures ensure consistent accuracy across varying environmental conditions. Temperature variations can impact pyranometer measurements by affecting the sensitivity and electrical output of the sensor. In colder conditions, the sensor's response might be less sensitive, leading to underestimation of solar irradiance. Conversely, in hotter conditions, the sensor might over-respond, leading to overestimations. ISO 9847 standards recommend procedures for compensating these temperature effects during calibration, ensuring that pyranometers produce accurate readings across different environmental temperatures. Calibration factors derived from ISO 9847 standards enhance the long-term accuracy and stability of solar pyranometer measurements by standardizing calibration procedures, incorporating environmental condition adjustments like temperature compensation, and recommending regular recalibration. This ensures consistent and reliable performance of pyranometers across varying environmental conditions.

**Table 1:** Calibration factors determined for each solar pyranometer.

Solar pyranometer number	Calibration factor (mV m^2^/ W)
1	13.38
2	11.67
3 (reference)	10.98
4	13.85
5	15.20
6	11.29

**Fig. 8:**
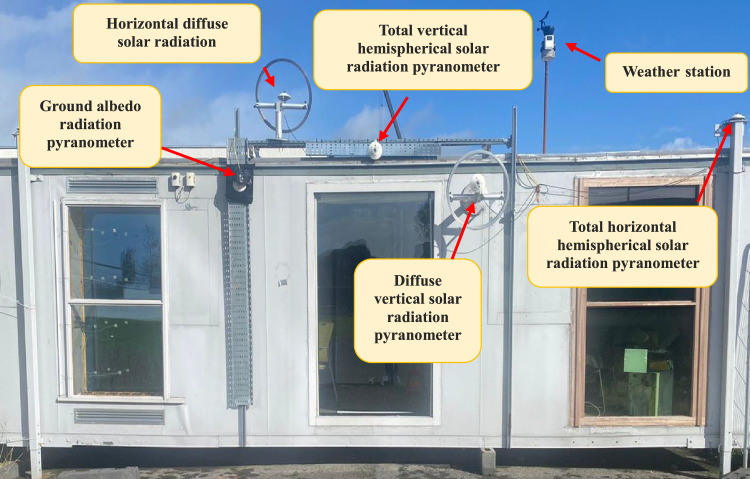
Pyranometers' locations on the facade.

After calibration, the solar pyranometers were mounted as shown in Fig. 8 on the south façade to measure (i) vertical total and diffuse solar irradiance, (ii) horizontal total and diffuse solar irradiance and (iii) ground reflected solar irradiance (albedo). Diffuse solar radiation was measured employing a shading ring, and the ground albedo was measured using a cover that blocked direct and diffuse sun irradiance.

## Limitations

Not applicable.

## Ethics Statement

All authors have read and follow the *ethical requirements* for publication in Data in Brief and confirming that the current work does not involve human subjects, animal experiments, or any data collected from social media platforms.

## CRediT authorship contribution statement

**H. Alkhatib:** Conceptualization, Methodology, Investigation, Visualization, Writing – original draft, Writing – review & editing. **B. Norton:** Supervision, Validation, Conceptualization, Methodology. **D.T.J. O'Sullivan:** Supervision, Validation, Conceptualization, Methodology. **P. Lemarchand:** Conceptualization, Methodology, Supervision, Validation, Software.

## Data Availability

Mendeley DataCalibration of Solar Pyranometers for Accurate Solar Irradiance Measurements: A Data Set from the Pre- and Post-Calibration Process. (Original data).
